# A randomized, double-blind, phase III, non-inferiority clinical trial comparing the efficacy and safety of TA4415V (a proposed Trastuzumab biosimilar) and Herceptin (Trastuzumab reference product) in HER2-positive early-stage breast Cancer patients

**DOI:** 10.1186/s40360-022-00599-x

**Published:** 2022-07-28

**Authors:** Reza Safaei Nodehi, Behjat Kalantari, Jahangir Raafat, Nafiseh Ansarinejad, Vahid Moazed, Seyed Mohammad Reza Mortazavizadeh, Mehran Hosseinzadeh, Bayazid Ghaderi, Arash Jenabian, Mojtaba Qadyani, Shirin Haghighat, Abolghasem Allahyari, Mehrzad Mirzania, Mohammad Seghatoleslami, Mehrdad Payandeh, Afsaneh Alikhasi, Hamidreza Kafi, Farhad Shahi

**Affiliations:** 1 Department of Internal Medicine, Hematology and Medical Oncology Ward, Cancer Research Center, Cancer Institute, Imam Khomeini Hospital Complex, Tehran University of Medical Sciences, Tehran, Iran; 2 Department of Internal Medicine, School of Medicine, Kerman University of Medical Sciences, Kerman, Iran; 3 Cancer Research Institute, School of Medicine, Tehran University of Medical Sciences, Tehran, Iran; 4 Iran University of Medical Sciences, Tehran, Iran; 5 Department of Internal, Faculty of Medicine, Islamic Azad University, Yazd, Iran; 6 Jundishapour University of Medical Sciences, Ahvaz, Iran; 7 Liver and Digestive Research Center, Kurdistan University of Medical Sciences, Sanandaj, Iran; 8 Department of Medical Oncology and Hematology, Boali Hospital, Tehran Medical Sciences, Islamic Azad University, Tehran, Iran; 9 Taleghani Hospital, Shaheed Beheshti University of Medical Sciences, Tehran, Iran; 10 Hematology Research Center, Department of Hematology, Medical Oncology, and Stem Cell Transplantation, Shiraz University of Medical Sciences, Shiraz, Iran; 11 Division of Hematology and Medical Oncology, Emam Reza Hospital, Mashhad University of Medical Sciences, Mashhad, Iran; 12 Hematology and Medical Oncology Department, Cancer Institute, Tehran University of Medical Sciences, Tehran, Iran; 13 Health Research Institute, Research Center of Thalassemia & Hemoglobinopathy, Ahvaz Jundishapur University of Medical Sciences, Ahvaz, Iran; 14 Regenerative Medicine Research Center, Kermanshah University of Medical Sciences, Kermanshah, Iran; 15 Department of Radiology, Cancer Institute of Iran, Tehran University of Medical Sciences, Tehran, Iran; 16 Medical Department, Orchid Pharmed Company, Tehran, Iran

**Keywords:** Breast Cancer, Trastuzumab, biosimilar, Non-inferiority, Randomized clinical trial

## Abstract

**Background:**

This study compared efficacy and safety of TA4415V, a trastuzumab biosimilar, with reference trastuzumab in patients with human epidermal growth factor receptor 2*–*positive (HER2-positive) early-stage breast cancer treated in the neoadjuvant setting in Iran.

**Methods:**

Patients were randomly assigned to receive neoadjuvant TA4415V or reference trastuzumab concurrently with docetaxel (TH phase) for 4 cycles after treatment with 4 cycles of doxorubicin and cyclophosphamide (AC phase). Chemotherapy was followed by surgery. The primary endpoint was the comparison of pathologic complete response (pCR) rate in the per-protocol population. Secondary endpoints included comparisons of overall response rate (ORR), breast-conserving surgery (BCS), safety, and immunogenicity.

**Results:**

Ninety-two participants were analyzed in the per-protocol population (TA4415V, *n* = 48; reference trastuzumab, *n* = 44). The pCR rates were 37.50% and 34.09% with TA4415V and reference drug, respectively. The 95% CI of the estimated treatment outcome difference (− 0·03 [95% CI − 0.23 to 0.16]) was within the non-inferiority margin. No statistically significant difference was observed between the groups for other efficacy variables in the ITT population: ORR (89.13% vs. 83.33%; *p* = 0.72) and BCS (20.37% vs. 12.96%; *p* = 0.42) in the TA4415V and reference drug group, respectively.

At least one grade 3 or 4 adverse events occurred in 27 (50%) patients in the TA4415V group versus 29 (53.70%) in the reference trastuzumab group (*p* = 0.70). The decrease in left ventricular ejection fraction (LVEF), as an adverse event of special interest (AESI) for trastuzumab, was compared between treatment groups in TH phase. Results demonstrated an LVEF decrease in 7 (12.96%) and 9 (16.67%) patients in TA4415V and reference trastuzumab groups, respectively (*p* = 0.59). Anti-drug antibodies (ADA) were not detected in any samples of groups.

**Conclusions:**

Non-inferiority for efficacy was demonstrated between TA4415V and Herceptin based on the ratio of pCR rates in HER2-positive early breast cancer patients. In addition, ORR and BCS, as secondary endpoints, were not significantly different. Safety profile and immunogenicity were also comparable between the two groups*.*

**Supplementary Information:**

The online version contains supplementary material available at 10.1186/s40360-022-00599-x.

## Background

Breast cancer is the most common malignancy worldwide and the leading cause of death due to cancer among women [1]. According to Institute for Health Metrics and Evaluation (IHME), there are more than 138,000 breast cancer cases in Iran [2].

Breast cancer is usually cured with a multidisciplinary approach involving surgery, radiation, and medical oncology, with systemic chemotherapy being an essential part of treatment [1, 3]. Human epidermal growth factor receptor 2 (HER2) protein overexpression is present in approximately 20% of breast cancer cases, resulting in a more aggressive natural subtype of the disease, poor prognosis [4], higher relapse, and higher mortality rates [5]. Patients with HER2-positive tumors benefit from adding anti-HER2 drugs to a cytotoxic chemotherapy backbone [6].

Neoadjuvant chemotherapy often results in tumor shrinkage, earlier treatment of subclinical distant micrometastases [7], and the facilitation of surgery. It may also increase rates of pathologic complete response (pCR) and favorable survival outcomes [8].

Trastuzumab is a humanized monoclonal antibody that binds to domain IV of the extracellular domain of the HER2 protein. This drug was the first Food and Drug Administration (FDA)-approved targeted therapy administered to treat HER2-positive breast cancer [9].

Trastuzumab combination with chemotherapy results in improvement of treatment response, progression-free survival (PFS), and overall survival (OS) in HER2-positive metastatic and early-stage breast cancer [10–12]. This addition also improved survival in HER2-positive metastatic gastric cancer patients compared to chemotherapy alone [13].

The European Medicines Agency (EMA) defines biosimilar as a biological medicine that is highly similar to another already approved biological medicine, known as reference medicine, with no clinically meaningful differences in terms of safety, purity, and efficacy [14]. Nowadays, biosimilars are vital components in treating serious diseases, including cancer, autoimmune diseases, and diabetes. Regarding poor facilities and limited access to reference medicines in developing countries [15], including Iran, it is imperative to provide citizens with low-cost biosimilar drugs [16].

TA4415V (AryoGen Pharmed, Iran) is a proposed trastuzumab biosimilar with structural and physicochemical characteristics similar to those of the trastuzumab reference product (Herceptin, Genentech, USA). AryoGen Pharmed is a high-tech company manufacturing biosimilar drugs in Iran [17, 18], which has achieved EU GMP certificate [17]. In addition, considering the price of Herceptin 440 mg and TA4415V 440 mg, it will reduce approximately 31% of trastuzumab costs for patients. At the time of the study, there was no obligation for performing preclinical studies for biosimilars according to Iran medicine agency. Preclinical assessments are required whenever a new drug formulation is developed.

The present study is a phase III clinical trial aimed to compare the efficacy, safety, and immunogenicity of TA4415V (registered as AryoTrust in Iran) with the reference drug (Herceptin, Genentech, USA) in HER2-positive early-stage breast cancer patients.

## Methods

### Study design

A phase III, randomized, two-armed, parallel, double-blind, active-controlled, non-inferiority clinical trial was conducted to compare the efficacy and safety of TA4415V with reference trastuzumab in HER2-positive breast cancer patients. The patients, research investigator, and data analyzer were blinded to the treatment assignments. Random assignment to treatment conditions was conducted by block randomization (block size of four) to either TA4415V or the reference product in a neoadjuvant setting.

This study was conducted according to the ethical principles of the declaration of Helsinki, Good Clinical Practice (GCP), local, ethical, and legal requirements. Both the study and clinical protocols were reviewed and approved by the ethics committee of Tehran University of medical sciences (IR.TUMS.REC.1395.2730) and Isfahan University of Medical Sciences (IR.MUI.REC.1395.4.041). The study has been registered in Iran Registry of Clinical Trials (IRCT201606226135N7) and Clinicaltrials.gov (NCT03425656).

### Intervention

Each patient was to receive 4 cycles of doxorubicin plus cyclophosphamide (AC), followed by 4 cycles of docetaxel plus trastuzumab (TH) as neoadjuvant treatment before undergoing surgery. In the AC phase, all patients received the same intravenous chemotherapy regimen (consisting of doxorubicin 60 mg/m^2^ plus cyclophosphamide 600 mg/m^2^) every 2 weeks. In the TH phase, each patient received docetaxel 100 mg/m^2^ and 8 mg/kg trastuzumab as a loading dose, followed by maintenance doses of 6 mg/kg administered every 3 weeks. Whether the patients should receive trastuzumab in the adjuvant setting after surgery or not, was based on the physician’s decision.

### Participants

#### Inclusion criteria

Eligible patients were women aged 18–70 years with a pathological diagnosis of breast adenocarcinoma. Patients were required to have newly diagnosed stage III or inoperable stage II with HER2-positive tumors (Immunohistochemical (IHC) 3+ intensity, or positive results of the HER2 gene on fluorescence or chromogenic in situ hybridizations (FISH or CISH)). Additional eligibility criteria were as follows: documented estrogen receptor (ER) and progesterone receptor (PR) status (positive or negative) and an Eastern Cooperative Oncology Group (ECOG) performance status of 0 or 1. Patients also had to be willing and able to sign informed consent.

#### Exclusion criteria

Exclusion criteria included clinical or radiologic evidence of metastatic breast cancer, history of any other malignancy including previous breast cancer, second non-breast malignant disease, and a history of previous chemotherapy. Other exclusion criteria were left ventricular ejection fraction (LVEF) < 55% (confirmed by an echocardiogram within 3 months before registration), any prior myocardial infarction, history of documented congestive heart failure (CHF), history of arrhythmia or cardiac valvular disease requiring medications or that were otherwise clinically significant, current use of medications for angina pectoris, current uncontrolled hypertension (diastolic> 100 mmHg or systolic> 200 mmHg), severe conduction abnormality (having a pacemaker or diagnosed by the electrocardiogram), and any other significant cardiovascular disease. Further exclusion criteria were hematologic abnormalities, liver and renal dysfunctions at baseline, pregnancy, lactation, and women of childbearing potential who were unwilling to use adequate contraception.

### Outcomes

The primary objective of the present study was to assess the non-inferiority of TA4415V in comparison to reference trastuzumab in terms of pCR. pCR was defined as “the absence of residual invasive cancer on hematoxylin and eosin evaluation of the complete resected breast specimen and all sampled regional lymph nodes following completion of neoadjuvant systemic therapy” [19] which is ypT0/is ypN0. Additionally, breast pathologic complete response (bpCR), which is defined as the absence of invasive neoplastic cells at the microscopic examination of the primary breast tumor at surgery (ypT0/is), was assessed in both treatment groups [20]. Surgery procedures were performed similarly in all centers; moreover, the methodology and references used for pCR assessments were the same among all pathologists. Only one surgeon and one pathologist from each center were involved in the study.

Secondary outcomes were related to comparisons of the overall response rate (ORR) and BCS in the two arms. ORR is defined as clinical complete response (cCR) or clinical partial response (cPR) as assessed based on the Response Evaluation Criteria in Solid Tumours version 1.1 (RECIST 1.1) [21]. Tumor assessments were conducted during the screening visit—after the AC phase, at the end of the neoadjuvant period (after TH phase) by sonography, magnetic resonance imaging (MRI), or mammography. ORR was measured at the end of the TH phase (before surgery) according to a blinded central review. BCS applicability was assessed in surgery visit, and the proportion of patients who underwent BCS was calculated.

Safety was assessed as another secondary outcome throughout the trial and the safety population was defined as all patients who received at least one dose of the treatment. Safety endpoints included the incidence and severity of adverse events (AEs), laboratory measures, and vital sign measurements. AEs were ranked according to the Common Terminology Criteria for Adverse Events (CTCAE), version 5.0 [22]. Treatment adverse events were summarized by study periods: AEs in AC, ACTH and TH phase. Causality assessment was performed according to WHO causality assessment scale.

For immunogenicity assessment, samples were collected before the first, second, and fourth trastuzumab administrations and 3 weeks after the surgery visit. The enzyme-linked immunosorbent assay (ELISA) method was conducted for anti-drug antibody (ADA) measurement.

### Statistical analysis

The study’s hypothesis was that TA4415V was non-inferior to reference trastuzumab. The non-inferiority margin was considered 25% of the pCR rate difference in reference trastuzumab compared to TA4415V. A total sample size of 108 (54 patients in each arm) with a 10% dropout rate was calculated based on the 60% pCR rate in the reference trastuzumab group and an expected 35% pCR rate in the TA4415V group, thus providing 80% power and the significance level of 5%. 60% pCR rate was defined based on a similar study and investigators’ opinion [23]. The statistic test used is the one-sided Z test (unpooled). The null hypothesis was that the upper limit of the rate difference in patients with pCR in the reference trastuzumab and TA4415V proceed 25%.

The intention-to-treat (ITT) population was defined as all patients randomly allocated to the study drug, regardless of whether a dose of the study drug was received. The per-protocol (PP) population comprised all patients in the ITT population except those who had a major protocol deviation.

The primary outcome was first evaluated in the PP population and then in the ITT population (with and without imputation) as a sensitivity analysis. In the imputation for pCR, patients with no available data were considered as nonresponders.

A two-sided 95% confidence interval for the difference between pCRs was calculated based on the proportion test with no adjustment for covariates. For the secondary efficacy analysis, we analyzed the overall response and BCS rate using descriptive statistics, which were then compared between groups using either a chi-square test or Fisher exact test.

The safety set was defined as all patients who received at least one dose of the study drug. AEs were categorized by study period as follows: AEs in the AC phase (defined as AEs that occurred and were resolved while the patient was treated with Doxorubicin and Cyclophosphamide), AEs in the TH phase (AEs that occurred from cycle 5 and onward while the patient received Docetaxel and Trastuzumab), and AEs in the ACTH phase (AEs that occurred while the patient was treated with AC but continued into TH phase).

For all statistical analyses, *p* < 0.05 was considered statistically significant. Also, statistical analysis was performed using STATA software (version 14, StataCorp LP, USA) and Rstudio software (RStudio Inc., USA).

## Results

**Fig. 1:**
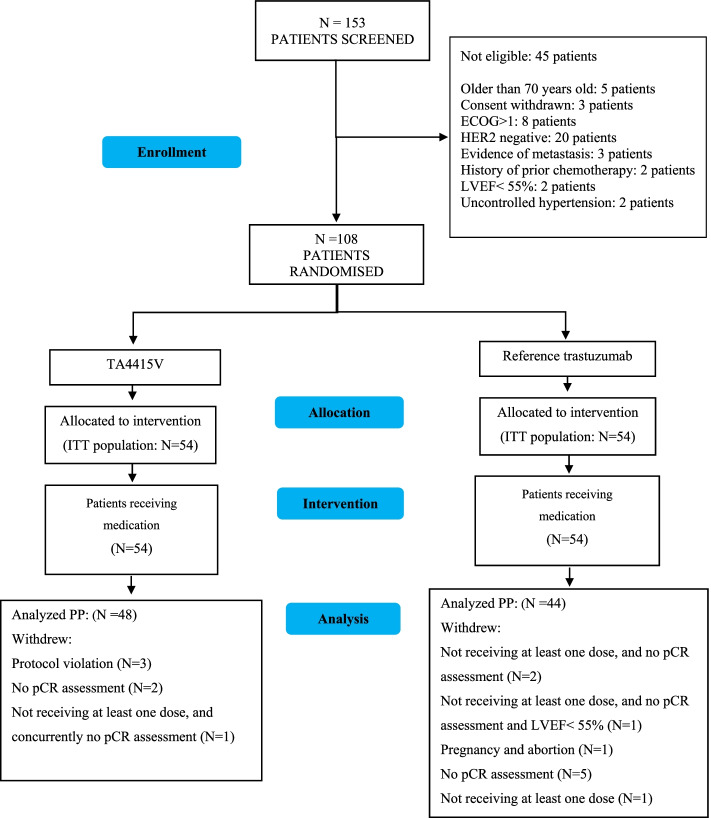
Patient disposition

Between July 2016 and August 2018, of the 153 patients screened, we recruited 108 patients with a 1:1 allocation. This trial was operated as a multi-center trial in 10 cities in Iran. Patient disposition is demonstrated in Fig. 1.

Demographics and baseline characteristics were balanced across treatment groups, with no meaningful differences between the two arms (Table 1).

**Table 1:** Baseline Characteristics of the participants in the Intention-to-Treat Population

	TA4415V (***N*** = 54)	Reference trastuzumab (***N*** = 54)
**Age (Years)**		
Median (range)	47.5 (28, 63)	46 (26, 69)
**Weight (Kg)**		
Median (range)	70 (46, 110)	70 (44, 100)
**Tumor Size (mm)**		
Median (range)	33(22,44)	33(21,41)
**IHC 2+,** No. (%)	9 (16.67)	11 (20.37)
**FISH+,** No. (% of IHC 2+)	3 (33.33)	2 (18.18)
**CISH+,** No. (% of IHC 2+)	6 (66.67)	9 (81.82)
**IHC 3+,** No. (%)	45 (83.33)	43 (79.63)
**ER/PR**		
ER and/or PR positive	36 (66.67)	30 (55.56)
ER and PR negative	18 (33.33)	24 (44.44)

There is no imputation for missing values

*IHC* Immunohistochemistry, *FISH* Fluorescence in situ hybridization, *CISH* Chromogenic in situ hybridization, *ER* Estrogen receptor, *PR* Progesterone receptor

### Efficacy

#### Primary outcome

The primary outcome (pCR) was analyzed according to the ITT and PP population, and non-inferiority was concluded through the PP population. The pCR rates in the PP population were 37.50% (18 patients) in the AryoGen trastuzumab group and 34.09% (15 patients) in the reference trastuzumab group (Table 2). Therefore, with a mean difference of − 0.03 (− 0.23, 0.16) and a 95% confidence interval for difference, TA4415V is considered to be non-inferior to reference trastuzumab.

**Table 2:** pCR analyses in the PP and ITT populations

	TA4415V (***N*** = 54)	Reference trastuzumab (***N*** = 54)	Difference (95% CI)
**Per-Protocol Population**^**a**^			
**pCR**	18 (37.50)	15 (34.09)	−0.03 (− 0.23, 0.16)
**Intention-to-treat Population**			
**pCR**^b^	19 (37.25)	16 (34.78)	−0.02 (− 0.22, 0.17)
**pCR**^**c**^ **(**Imputed analysis**)**	19 (35.19)	16 (29.63)	−0.05 (− 0.23, 0.12)

Data are n (%), unless otherwise indicated

Difference = proportion (reference trastuzumab) – proportion (TA4415V)

^a^*n* = 48 for TA4415V and *n* = 44 for reference trastuzumab

^b^*n* = 51 for TA4415V and 46 for reference trastuzumab

^c^*n* = 54 for TA4415V and 54 for reference trastuzumab

Sensitivity analyses in the ITT population were planned, with and without imputation, to assess the robustness of the main results. For analysis with imputation, 11 patients with no pCR data were considered as non-pCR. The results in the ITT population were similar to those in the PP population (*p* = 0.80). These data are presented in Table 2.

**Fig. 2:**
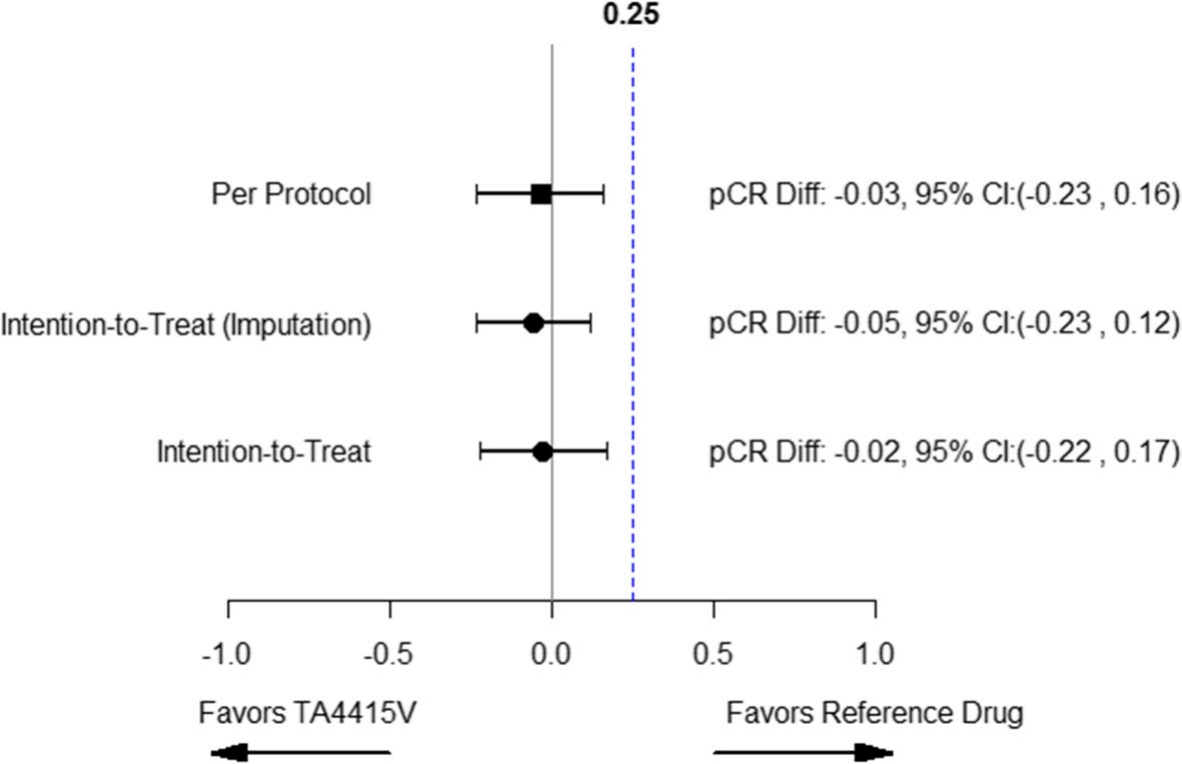
Forest plot for pCR result of PP and ITT analysis

The forest plot of pCR was plotted for both treatment populations and is demonstrated in Fig. 2. According to this figure, the upper limit of 95% CI of the pCR difference was lower than the prespecified non-inferiority margin (0.25) in two population sets (ITT and PP).

Among patients with negative hormone receptor status, 47.06% of 51 and 40.91% of 46 participants had pCR in the TA4415V and reference trastuzumab groups, respectively. In hormone receptor-positive patients, 32.35% showed a pCR in the TA4415V, compared with 29.17% in the reference trastuzumab group.

#### Secondary outcomes

##### bpCR

bpCR was analyzed as the secondary outcome in ITT population. bpCR rate was 39.22% (20 patients) in TA4415V and 45.65% (21 patients) in the reference trastuzumab group. The difference between the two groups was not statistically significant (*P*-value = 0.52).

##### ORR

ORR was 89.13% in the TA4415V arm with 15.22% of participants showing CR and 73.91% showing PR. The proportion of patients with ORR was 83.33% in the reference trastuzumab group (CR: 23.81%, PR: 59.52%). All clinical responses in the treatment groups are presented in Table 3. There was no statistically significant difference in the ORR rate among groups (*p* = 0.72).

**Table 3:** Proportion of Clinical Response in the treatment groups

	TA4415V	Reference trastuzumab
**Complete Response (%)**	15.22	23.81
**Partial Response (%)**	73.91	59.52
**Progressive Disease (%)**	0.00	2.38
**Stable Disease (%)**	6.52	9.52
**Unassessable (%)**	4.35	4.76

Valid percent was reported

*P*-value = 0.59

*P*-value for ORR (Overall response includes complete and partial response) = 0.72

*P*-value computed using fisher exact Test

##### BCS

Seven patients (12.96%) in the reference trastuzumab group and 11 patients (20.37%) in the TA4415V group underwent BCS. The difference was not statistically significant among these groups (*p* = 0.42).

Among 79 patients who had a mastectomy (TA4415V: 40, reference trastuzumab: 39), 13 patients in the TA4415V and 12 patients in the reference trastuzumab group achieved pCR.

### Safety

#### Adverse events

According to the analysis, 34.28%, 6.92%, and 58.81% of AEs were observed in AC, ACTH, and TH phases, respectively. Among these, 534 AEs occurred in the reference trastuzumab group, and 588 AEs were reported in the TA4415V group. No deaths were reported during the study, and the only serious adverse event was an abortion due to the physician’s decision, but pregnancy was not reported to the sponsor at the time. This event occurred in the AC phase and before the administration of reference trastuzumab.

The severity levels of most events were mild or moderate in both treatment groups. Incidence of grade 3 and 4 AE was also reported, and there was no significant difference in severity among the two groups (*p* = 0.70).

AEs that occurred in the AC phase were considered unlikely because trastuzumab, as the experimental drug, was not administered in this phase. At least possibly related AEs (possible or probable) were also reported in the ACTH and TH phase. Two AEs led to drug discontinuation: one case of LVEF decrease occurred in the AC phase, and one case of hepatitis occurred in the TH phase (Table 4).

**Table 4:** Adverse events in ITT population

	AC	TH		ACTH	
		TA4415V N (%)	Reference trastuzumab N (%)	TA4415V N (%)	Reference trastuzumab N (%)
**At least possibly related AE**	–	53 (98.15)	49 (90.74)	24 (44.44)	28 (51.85)
**Serious AE**	1 (0.93)	0 (0.00)	0 (0.00)	0 (0.00)	0 (0.00)
**Number of subjects reporting at least one grade 3 or 4 AE**	32 (29.63)	27 (50.00)	29 (53.70)	1 (1.85)	1 (1.85)
**AEs leading to drug discontinuation**	1 (0.93)	1 (1.85)	0 (0.00)	0 (0.00)	0 (0.00)

The decrease in ejection fraction, as the AE of special interest (AESI), was compared between treatment groups in the TH phase. An LVEF decrease was reported in seven patients (12.96%) and nine patients (16.67%) in the TA4415V and reference trastuzumab groups, respectively; no significant difference was observed between these groups (*p* = 0.59). Table 5 shows descriptive data for LVEF values at baseline, visit 5 and visit 9 for TA4415V (respectively 57.94 (56.91–59.03), 55.58 (56.72–58.43), 56.83 (55.40–58.26)) and Reference trastuzumab (respectively 56.92 (55.42–58.41, 55 (55–60), 55.02 (52.98–57.05)). Patients received TH regimen between visit 5 and visit 9.

**Table 5:** Descriptive Data for LVEF

Visit and Statistic	TA4415V		Reference trastuzumab	
Baseline		Change from Baseline		Change from Baseline
Mean (95% CI)	57.94 (56.91,59.03)		56.92 (55.42,58.41)	
Median (Range)	60 (55,60)		55 (55,60)	
Visit 5				
Mean (95% CI)	55.58 (56.72,58.43)	0.30 (− 0.92,1.52)	55.63 (54.10,57.16)	1.92 (− 0.02,3.896)
Median (Range)	58.25 (55,60)	0 (0,1)	55 (55,60)	0 (− 1.25,5)
Visit 9				
Mean (95% CI)	56.83 (55.40,58.26)	0.39 (−0.89,1.67)	55.02 (52.98,57.05)	1.39 (−0.54,5.22)
Median (Range)	57.5 (55,60)	0 (−2.5,2.5)	55 (55,57.5)	0 (−1,5)

#### Immunogenicity

Up to the end of the study, immunogenicity was negative in all obtained samples. Since there was no ADA emergence, the relationship between immunogenicity, treatment efficacy and safety could not be statistically analyzed.

## Discussion

The present study was a phase III, randomized, active-controlled trial designed to evaluate the non-inferiority of TA4415V (as a proposed biosimilar) versus the reference drug. Patients with early-stage and operable HER2-positive breast cancer received neoadjuvant treatment with trastuzumab as a part of their therapeutic regimen. This population is more suitable than metastatic patients for inclusion in studies intended to evaluate biosimilarity, due to their homogeneity, low tumor burden, and lower risk for disease progression and severe adverse events (SAEs) [24]. Considering the administration of trastuzumab in the neoadjuvant setting, the potential shortened exposure to trastuzumab must be taken in to account while interpreting the results. pCR was assessed as the primary endpoint, as the FDA and the EMA guidelines approve the use of pCR as a surrogate endpoint for the neoadjuvant treatment of high-risk, early-stage breast cancer [19, 25].

The proportion of HER2-positive breast cancer patients achieving pCR in the TA4415V group was statistically similar to patients treated with the reference drug, and the results confirmed non-inferiority. All secondary efficacy analyses (ORR and BCS) were comparable between groups, as well as their safety profiles.

The present study showed the therapeutic non-inferiority of biosimilar TA4415V to reference trastuzumab in patients with early-stage, operable, HER2-positive breast cancer. The pCR rates are consistent with those reported in previous randomized controlled trials on trastuzumab therapy for HER2-positive breast cancer patients. Similar findings were reported in a GeparQuattro study, in which trastuzumab was added to neoadjuvant chemotherapy for patients with operable or locally advanced HER2-positive breast cancer. This treatment resulted in a 31.7% pCR rate, which was 16% higher than that in the chemotherapy alone group [26]. Similarly, in a NOAH study, patients receiving trastuzumab plus chemotherapy achieved a pCR rate of 39% [27]. In another study conducted by Untch et al. receiving trastuzumab with chemotherapy for the neoadjuvant treatment of HER2-positive breast cancer patients resulted in 39% pCR [8]. In addition, the study of neoadjuvant PF-05280014, an FDA-approved biosimilar trastuzumab, with docetaxel and carboplatin by Lammers et al., for operable HER2-positive breast cancer patients, showed 47.0% pCR [28]. In the LILIAC study, receiving ABP 980, as another trastuzumab biosimilar, in patients with HER2-positive early breast cancer, resulted in 48% pCR [29].

Clinical trials comparing the safety and efficacy of trastuzumab biosimilars and evaluating pCR and ORR in patients with early and metastatic breast cancer are ongoing [30]. In a study from pivot et al. ERBB2-positive early breast cancer patients received HD201 (Trastuzumab biosimilar) and Referent Trastuzumab in a neoadjuvant setting for 8 cycles concurrently with chemotherapy, and tpCR rates were 45% for HD201 and 48.7% for referent trastuzumab [31]. In another study of Pivot et al., breast cancer patients received neoadjuvant trastuzumab for 8 cycles concurrently with chemotherapy, and a pCR rate of 35.8% was reported in the trastuzumab biosimilar group, which is close to the pCR rate in our study [32]. This rate was reported to be higher (46.8% in the biosimilar group and 50.4% in the reference group) in a study by Stebbing et al. Patients in this trial received trastuzumab in combination with chemotherapy for 8 cycles, followed by surgery. The results demonstrate that pCR rates are higher in hormone receptor-negative patients. In the present study, patients were not stratified according to hormone receptor status, and the difference in pCR rates with other studies may be a result of high number of ER/PR positive patients. Breast pCR was also assessed in this trial, and the results were similar to those of the present study, with a bpCR rate of 51.6% [33]. The most similar result to the present study was the 39.7% pCR rate reported in the trastuzumab group [32].

A limitation of our study is that random assignment was not stratified by hormone receptor status, even though this factor could influence treatment response. The prespecified margin was defined based on an article along with the investigators’ opinion [23]. Moreover, based on the study protocol, this trial was performed in the neoadjuvant setting only. Therefore, the potential adjuvant treatment, post-surgery follow-ups and survival outcomes were not assessed. The follow-up duration for safety assessment was also short.

Another limitation of the study was that 60% pCR rate was defined based on the clinical judgment along with the results of a study on that time [23]. Considering the different potential definitions of response in our trial and physician’s routine practice, it seems that the prespecified threshold was overestimated. As the pCR rate in the control arm was lower than the assumption of 60% which is consistent with some other studies previously explained. We considered a 25% non-inferiority margin based on the assumption of 60% pCR rate. However, considering the forest plot regarding the pCR rate results in the study (Fig. 2), non-inferiority is still achievable by considering 16% margin in the per protocol population and 17% margin in the ITT population without imputation. Non-inferiority was concluded based on the calculated confidence interval although larger sample size causes a narrower confidence interval.

Pre-surgery ORR was evaluated as a secondary endpoint in this study. Different trials reported varying ORR values, ranging from 87% [27, 33] to 96% [32]. In the study of Lammers et al. operable HER2-positive patients receiving neoadjuvant PF-05280014, showed 88.1% ORR [28]. The closest ORR to our study among all trials was reported by Lammers et al.; specifically, the ORR rate was 88.1% before surgery [28].

Trastuzumab administration in patients with HER2-positive disease does not necessarily increase lumpectomy. In the clinical setting, several factors influence the choice of surgery type after neoadjuvant therapy. For tumors larger than 5 cm, ER-negative, multicentric or multifocal, conservative surgery is less likely. However, the two most important factors influencing surgery choice are the surgeon’s recommendation at diagnosis and the patient’s personal preference. Despite achieving a pCR, surgeons might still apply a more aggressive approach and recommend a mastectomy. In addition, patients tend to stay with the original pre-chemotherapy mastectomy recommendation, even if the treatment response is excellent [34–36]. The closest rate of lumpectomy to the present study was 20.3%, which was associated with another trastuzumab biosimilar comparable to reference trastuzumab (22.6%) [33].

The safety profiles of TA4415V and the reference drug during the neoadjuvant period were not statistically different, in line with other trials [27–30]. No unexpected adverse events occurred in the treatment groups, and no deaths were reported throughout the study. The only serious adverse event was one abortion, which occurred in the AC phase, and, therefore, was not related to the drugs used in the study. There were also two AEs that led to drug discontinuation, one case of LVEF decrease, and one case of hepatitis. The rate of cardiovascular events, as an adverse event of special interest (AESI) for trastuzumab, was 10% in the reference trastuzumab group in Stebbing et al.’s study, which is similar to the present study [33].

Immunogenicity was negative in both treatment groups, indicating the low immunogenic potential of both drugs. This result is consistent with previous studies that used the intravenous administration of trastuzumab (0–3.4%) [28, 29, 33, 37].

## Conclusion

TA4415V (biosimilar trastuzumab) was non-inferior in efficacy and comparable in safety to Herceptin in the treatment of HER2-positive breast cancer.

## Supplementary Information

**Additional file 1.**

## Data Availability

The datasets generated and/or analysed during the current study are not publicly available due to the consent form but are available from the corresponding author on reasonable request.
