# Supplementary material for: A randomized, double-blind, phase III, non-inferiority clinical trial comparing the efficacy and safety of TA4415V (a proposed Trastuzumab biosimilar) and Herceptin (Trastuzumab reference product) in HER2-positive early-stage breast Cancer patients
Source: BMC Pharmacol Toxicol. 2022 Jul 28;23:57. doi: 10.1186/s40360-022-00599-x (PMC9336069; doi:10.1186/s40360-022-00599-x)
Supplement: Supplementary file 1 — Additional file 1. [file 40360_2022_599_MOESM1_ESM.docx]

Summary of immunogenicity study

(AryoTrust **^TM^** lyophilized powder for preparation Concentrated solution for intravenous infusion)

**AryoGen Pharmed**

No. 140, 24th Km Tehran-Karaj Makhsous road, Alborz, Iran

1. **Summary of procedure**

The procedure is ELISA. In this method, the LISA-TRACKER Trastuzumab kit from Theradiag company was used to measure the amount of antibody against Trastuzumab. This kit uses plates with Trastuzumab coated on the bottom and it is ready.

The steps are as follows:

- The patient sample is mixed with 300 mM acetic acid in a ratio of 1 to 10 and the diluted samples are placed at 23±2˚ C for one hour.
- After one hour of incubation of the samples, according to the instruction, 30µl Tris buffer (pH = 9.5 ± 0.1) is added to all plate wells except the standard kit wells and the positive sample.
- 100µl of acid-diluted samples are added to each well and placed at 23 ± 2˚C for 3 hours.
- Since standard kit samples must be incubated for one hour, after two hours of incubation of the diluted samples with acid, standard samples are added to the respective wells and stored for one hour at 23 ± 2˚C is inserted.
- The wells are washed 4 times with ~ 300 µl of TDL buffer.
- 100 µl of Biotinylated antibody is added to each well.
- The plate is incubated for 1 hour at room temperature (23 ± 2 ° C).
- The wells are washed 4 times with ~ 300 µl of TDL buffer.
- 100 µl of HRP labeled Streptavidin is added to each well.
- The plate is incubated for 30 minutes at room temperature (23 ± 2 ° C).
- The wells are washed 3 times with ~ 300 µl of TDL buffer.
- 100 µl of TMB is added to each of the wells.
- The plate is incubated for 15 minutes at room temperature (23 ± 2 ° C).
- 100 µl of stop solution is added to each of the wells.
- Light absorption of wells is measured at a wavelength of 450 nm.

1. **System suitability**

- Kit standards including 0 (blank), 10.0, 40.0, 80.0, 120.0 ng / ml of Anti-Trastuzumab are analyzed and the calibration line should be R2 = 0.99.
- RSD% of repetitions must be below 10.0%.
- Highest OD to lowest OD must be greater than 5.0.

Table1. System suitability

| Concentration | Rep 1 | Rep 2 | Rep 3 | Average |
| --- | --- | --- | --- | --- |
| 120 | 0.993 | 0.726 | 0.546 | 0.755 |
| 80 | 0.737 | 0.443 | 0.434 | 0.538 |
| 40 | 0.317 | 0.324 | 0.218 | 0.286 |
| 10 | 0.139 | 0.090 | 0.097 | 0.109 |
| 0 | 0.098 | 0.086 | 0.088 | 0.091 |

1. **Data analysis**

In each plate, the amount of Cut-Off is obtained for each plate as follows:

$$Cut-Off=Mean of Negative Controls \times2$$

If the OD of the sample obtained from the cut-off is higher in each plate, the respective sample is reported as positive.

The average cut-off of the samples is 0.21, which is about 25.0 - 30.0 ng / ml of anti-trastusumab antibody.

1. **Validation, accuracy and QA and QC methods**

Validity and accuracy: To ensure the answers of this method, the method has been validated and its validation has been checked for the accuracy of the reported answers and its documents are available in the quality assurance section with this number of documents:

| **Title** | **Validation Number** |
| --- | --- |
| Measurement of anti-Trastuzumab antibodies in Human serum samples using ELISA method (Validation Protocol) | VP-AD-106 |
| Measurement of anti-Trastuzumab antibodies in Human serum samples using ELISA method (Validation Report ) | VR-AD-107 |
| Measurement of Anti-Trastuzumab Antibodies in Human Serum Samples Using ELISA Method (SOP) | QC-07-05-014 |
| Measurement of Anti-Trastuzumab Antibodies in Human Serum Samples Using ELISA Method( Datasheet) | QC-7087 |

This test is performed based on the relevant SOP mentioned above and the data of this test are recorded in the relevant register and archived in quality assurance.

1. **The final conclusion**

Out of 282 samples analyzed, the level of antibodies against trastuzumab in serum was less than the permissible level and all of these samples were reported as negative.
